# Colorectal cancer chemotherapy: can sex-specific disparities impact on drug toxicities?

**DOI:** 10.1007/s00228-022-03298-y

**Published:** 2022-02-22

**Authors:** Silvia De Francia, Paola Berchialla, Tiziana Armando, Silvana Storto, Sarah Allegra, Veronica Sciannameo, Giulia Soave, Andrea Elio Sprio, Silvia Racca, Maria Rosaria Caiaffa, Libero Ciuffreda, Maria Valentina Mussa

**Affiliations:** 1 Department of Clinical and Biological Sciences, Clinical Pharmacology Service Franco Ghezzo, University of Turin, S. Luigi Gonzaga Hospital, Regione Gonzole 10, 10043 Orbassano, TO Italy; 2 Department of Public Health, University of Turin, Turin, Italy; 3 Unit of Biostatistics, Epidemiology and Public Health, Department of Cardiac, Thoracic, and Vascular Sciences and Public Health, University of Padova, Padova, Italy

**Keywords:** Sex, Personalized medicine, Adverse events, Chemotherapy, Colorectal cancer

## Abstract

**Purpose:**

Given the biological differences between females and males, sex-specific evaluations should be carried out to obtain better cancer prevention, diagnosis, and treatment strategies. To this purpose, our aim was to evaluate sex differences for toxicity in a cohort of colorectal cancer (CRC) patients undergoing chemotherapy.

**Methods:**

We performed a retrospective study in 329 CRC patients. Differences between males and females were tested performing the Mann-Whitney *U* test or the Fisher exact test. Multivariate logistic regression models were computed to evaluate the association between sex and risk of chemotherapy agent-related toxicity.

**Results:**

According association sex toxicity, significant differences were observed in the median number of episodes of nausea (*p* = 0.044), vomit (*p* = 0.007), heartburn (*p* = 0.022), thrombocytopenia (*p* = 0.005), mucositis (*p* = 0.024). Moreover, statistically significant differences between males and females were observed in the distribution of the highest toxicity grades of nausea (*p* = 0.024), heartburn (*p* = 0.016), and thrombocytopenia (*p* = 0.034). Females have an increased risk of vomit (*p* = 0.002), alopecia (*p* = 0.035), heartburn (*p* = 0.005), mucositis (*p* = 0.003), and lower risk for thrombocytopenia (*p* = 0.005).

**Conclusion:**

According to the association of sex chemotherapy agent-related toxicities, females resulted on average at a significant increased risk of more common adverse events (constipation, dysgeusia, alopecia, heartburn, vomit, asthenia, nausea, pain events, and mucositis). Sex-tailored CRC chemotherapy treatment is necessary to obtain efficacy avoiding toxicity, based on patients’ biological and genetic characteristics, a vision that would change CRC setting, a stable disease but still orphan of a real tailored approach.

**Supplementary information:**

The online version contains supplementary material available at 10.1007/s00228-022-03298-y.

## Introduction

Aside from gender-specific tumors (breast, prostate, ovarian cancer), recent studies demonstrate gender-specific incidence, progression, and severity of different tumors. Differences have to be investigated in a parameter such as body weight, fat distribution, hormonal profile, metabolism, immune response, and genetics [1, 2]. Among other physiological factors, different metabolic enzymes and specific liver and kidney transporters concur to modulate pharmacokinetics and pharmacodynamics according to gender. Women have a slower gastric emptying time and a bigger distribution volume for lipophilic drugs. Chemotherapeutical compounds show, thus, a 1.7-fold increase in adverse effects in women, although the longer half-life provides a benefit in terms of survival rate [3–8]. Analogously, women are more sensitive to toxicity, especially at the gastrointestinal and mucosal level, when treated with 5-fluorouracile. Moreover, nausea and vomit are increased because of the lower efficacy of antiemetic drugs [9, 10].

A gender analysis, therefore, is necessary for the equity of care in clinical settings such as oncology, where extensive and very toxic drugs are widely used [11–13]. Sex-specific analyses are of primary importance to establish targeted anti-cancer drug agents. In our study, we explored the effect of sex on reported toxicity in a cohort of colorectal cancer (CRC) patients undergoing different chemotherapy treatments, also considering concomitant administered drugs.

## Materials and methods

We performed a retrospective study of CRC-diagnosed patients, enrolled at the Medical Oncology Unit of the Molinette Hospital, AOU City of Health and Science of Turin, from October 2016 to July 2018. The database was prepared with collected data from the TrakCare® Hospital System Medical Records. Inclusion criteria were CRC diagnosis, undergoing active therapy, age ≥ 18 years old, and Day Hospital exclusive affiliation. The following variables were collected: sex, age, TNM classification, comorbidities, drugs taken at home and during chemotherapy treatment, chemotherapy cycle scheme, gastrointestinal toxicity (mucositis, nausea, vomiting, diarrhea, constipation), neurological toxicity (peripheral or central neuropathy), skin toxicity (alopecia, hand-foot syndrome), hematological toxicity (thrombocytopenia, neutropenia, anemia), other toxicities (asthenia, dysgeusia, epistaxis, fever, and hyporessia), pain, epigastralgia, recurrence, visual disturbances, proteinuria, hypertension, and hypotension. Common Terminology Criteria for Adverse Events (CTCAE version 4.0) scoring system was used to detect toxicities. Data were collected for each day of hospital access. Based on the treatment schemes, toxicities have been attributed to several chemotherapy agents at the same time.

In Supplementary Table 1, we reported information on treatment schemes (chemotherapy agents). Patients included had a stable disease and therefore on average characterized by low Eastern Cooperative Oncology Group (ECOG) performance status of 0 or 1.

Considering the statistical analysis, the age was described using mean and standard deviations (SD). Categorical variables (tumor classification, reported comorbidities, and type of chemotherapy agent) were described using frequencies and percentages. Gender differences in tumor classification, reported comorbidities, and type of chemotherapy agent were tested performing the Fisher exact test. The distribution of toxicity episodes per patient and the distribution of toxicity grades in the different sex group were evaluated using the Fisher exact test, considering all the therapy agents together. Multivariate logistic regression models, adjusted by age, were computed to evaluate the association between sex and risk of toxicity considering all the treatment and topoisomerase inhibitors, antimetabolite pyrimidine analogues, DNA binding drugs, and grow inhibitor monoclonal antibodies schemes separately. Since the analysis was carried out at the visit level, the Huber-White estimator was used to adjust the correlation between multiple observations on the same patient. Odds ratios (OR) and their 95% confidence intervals (95% CI) were reported. Firth’s correction was applied to reduce the bias of the estimates due to a small number of events. Statistical analyses were performed using R version 3.4.0. The level of significance was set at 0.05.

These data were routinely recorded during daily clinical practice as a quality assurance measure and in order to explore improvements in the quality of services. Ethics committee approval was not required but the research project was the same submitted to the local ethics committee (Prot. N° 0092030, approved). Confidentiality was guaranteed in data collection, analysis, and dissemination phase, by presenting the results in aggregate form.

## Results

### Study population

We enrolled 329 CRC diagnosed patients, for a total hospital accesses of 10,355 days. Demographics characteristics along with clinical and pharmacological information were reported in Table 1 stratified by sex. Statistically significant differences have been observed on the prevalence of metabolic (12.2% females vs 22.0% males, *p* = 0.028), endocrine (13.5% females vs 2.9% males, *p* = 0.001), and renal (3.8% females vs 13.9% males, *p* = 0.003) comorbidities. Describing chemotherapy regimens, statistically significant differences have been observed in the proportion of females and males treated with topoisomerase inhibitors (respectively 11.5% and 13.9%, *p* < 0.001), tyrosine kinase inhibitor (0.4% and 1.2%, *p* < 0.001), and antibiotics (0.5% and 0.1%, *p* = 0.002).

**Table 1:** Demographic, clinical, and pharmacological description of the enrolled patients

	*F*	*M*	*p*-value
Patients (*N* (%))	156 (47.42)	173 (52.58)	
Age (mean (SD))	65.16 (11.27)	66.31 (9.90)	0.331
Classification of malignant tumors, TNM (*N* (%))			0.585
T1	2 (1.3)	5 (2.9)	
T2	9 (5.8)	11 (6.4)	
T3	66 (42.3)	81 (46.8)	
T4	33 (21.2)	27 (15.6)	
N0	156 (47.42)	173 (52.58)	
M0	156 (47.42)	173 (52.58)	
Not reported	46 (29.5)	49 (28.3)	
**Comorbidities (*N* (%))**			
Cardiovascular	57 (36.5)	80 (46.2)	0.095
Metabolic	19 (12.2)	38 (22.0)	**0.028**
Former tumor	7 (4.5)	16 (9.2)	0.140
Endocrine	21 (13.5)	5 (2.9)	**0.001**
Respiratory	6 (3.8)	10 (5.8)	0.577
Neurologic and psychiatric	12 (7.7)	15 (8.7)	0.903
Gastrointestinal	23 (14.7)	17 (9.8)	0.233
Infectious diseases	11 (7.1)	8 (4.6)	0.480
Renal	6 (3.8)	24 (13.9)	**0.003**
Orthopedic	11 (7.1)	5 (2.9)	0.135
**Visits**	*N* = 4618	*N* = 5737	
Chemotherapy agents (*N* (%))			
Antimetabolite pyrimidine analogues	2199 (48.1)	2684 (47.5)	0.542
Topoisomerase inhibitors	528 (1.5)	787 (13.9)	**< 0.001**
DNA binding drugs	1136 (24.8)	1392 (24.6)	0.809
Growth inhibitor monoclonal antibody	1253 (27.4)	1550 (27.4)	1.000
Tyrosine kinase inhibitor	19 (0.4)	69 (1.2)	**< 0.001**
Mitotic inhibitors	13 (0.3)	13 (0.2)	0.730
Antibiotics	21 (0.5)	7 (0.1)	**0.002**
Antiestrogens	4572 (99.00)	5654 (98.55)	0.908
Bisphosphonates	1 (0.0)	3 (0.1)	0.772

In bold: statistically significant *p*-values (<0.05)

### Association between sex and toxicity

The distribution of the number of toxicity episodes per patient and of the highest toxicity grade stratified by sex is shown in Table 2. Statistically significant differences between females and males were observed in the median number of episodes of nausea (3 [IQR: 0–8] in females and 1 [IQR: 0–6] in males, *p* = 0.044), vomit (0 [IQR: 0–1] in females and 0 [IQR: 0–0] in males, *p* = 0.007), heartburn (0 [IQR: 0–1] in females and 0 [IQR: 0–0] in males, *p* = 0.022), thrombocytopenia (0 [IQR: 0–0] in females and 0 [IQR: 0–1] in males, *p* = 0.005), mucositis (0 [IQR: 0–2] in females, and 0 [IQR: 0–1] in males, *p* = 0.024).

**Table 2:** Number of episodes and grade distribution of toxicities

	*F*	*M*	*p*-value
	156	173	
**Nausea (*N*)**			
No. of episodes per patients (median [IQR])	3 [0,8]	1 [0,6]	**0.044**
Grade (*N* (%))			**0.024**
0	44 (28.2)	73 (42.2)	
1	39 (25.0)	45 (26.0)	
2	55 (35.3)	44 (25.4)	
3	18 (11.5)	10 (5.8)	
4	0 (0.0)	1 (0.6)	
**Vomit (retching) (*N*)**			
No. of episodes per patients (median [IQR])	0 [0,13]	0 [0,0]	**0.007**
Grade (*N* (%))			
0	99 (63.5)	131 (75.7)	0.06
1	25 (16.0)	22 (12.7)	
2	22 (14.1)	16 (9.2)	
3	10 (6.4)	4 (2.3)	
**Neurotoxicity (*N*)**			
No. of episodes per patients (median [IQR])	3 [0,8]	4 [0,11]	0.229
Grade (*N* (%))			
0	59 (37.8)	65 (37.6)	
1	27 (17.3)	39 (22.5)	0.613
2	62 (39.7)	61 (35.3)	
3	8 (5.1)	7 (4.0)	
4	0 (0.0)	1 (0.6)	
**Alopecia (*N*)**			
No. of episodes per patients (median [IQR])	0 [0,0]	0 [0,0]	0.075
Grade (*N* (%))			
0	124 (79.5)	150 (86.7)	
1	15 (9.6)	10 (5.8)	0.390
2	6 (3.8)	4 (2.3)	
3	5 (3.2)	6 (3.5)	
4	6 (3.8)	3 (1.7)	
**Asthenia (*N*)**			
No. of episodes per patients (median [IQR])	5 [1,1]	4 [0,9]	0.316
Grade (*N* (%))			
0	29 (18.6)	47 (27.2)	
1	47 (30.1)	48 (27.7)	0.177
2	53 (34.0)	44 (25.4)	
3	26 (16.7)	34 (19.7)	
4	1 (0.6)	0 (0.0)	
**Diarrhea (*N*)**			
No. of episodes per patients (median [IQR])	1 [0,4]	1 [0,5]	0.992
Grade (*N* (%))			
0	56 (35.9)	66 (38.2)	
1	30 (19.2)	34 (19.7)	0.944
2	45 (28.8)	43 (24.9)	
3	23 (14.7)	27 (15.6)	
4	2 (1.3)	3 (1.7)	
**Constipation (*N*)**			
No. of episodes per patients (median [IQR])	0 [0,1]	0 [0,1]	0.890
Grade (*N* (%))			
0	105 (67.3)	113 (65.3)	0.897
1	24 (15.4)	31 (17.9)	
2	21 (13.5)	24 (13.9)	
3	6 (3.8)	5 (2.9)	
**Pain (*N*)**			
No. of episodes per patients (median [IQR])	0 [0,2]	1 [1,0.2]	0.493
Grade (*N* (%))			
1	75 (48.1)	94 (54.3)	0.306
**Hand-foot syndrome (*N*)**			
No. of episodes per patients (median [IQR])	0 [0,3]	0 [0,5]	0.169
Grade (*N* (%))			
0	98 (62.8)	98 (56.6)	
1	21 (13.5)	17 (9.8)	0.156
2	17 (10.9)	20 (11.6)	
3	17 (10.9)	27 (16.6)	
4	3 (1.9)	11 (6.4)	
**Heartburn (*N*)**			
No. of episodes per patients (median [IQR])	0 [0,1]	0 [0,0]	**0.022**
Grade (*N* (%))			
0	102 (65.4)	132(76.3)	
1	33 (21.2)	24 (13.9)	**0.016**
2	21 (13.5)	13 (7.5)	
3	0 (0.0)	4 (2.3)	
**Dysgeusia (*N*)**			
No. of episodes per patients (median [IQR])	0 [0,2]	0 [0,2]	0.853
Grade (*N* (%))			
0	102 (65.4)	114 (65.9)	
1	24 (15.4)	31 (17.9)	0.584
2	28 (17.9)	23 (13.3)	
3	2 (1.3)	4 (2.3)	
4	0 (0.0)	1 (0.6)	
**Thrombocytopenia (*N*)**			
No. of episodes per patients (median [IQR])	0 [0,0]	0 [0,1]	**0.005**
Grade (*N* (%))			
0	129(82.7)	121(69.9)	
1	6 (3.8)	10 (5.8)	**0.034**
2	17 (10.9)	30 (17.3)	
3	3 (1.9.1)	12 (6.9)	
4	1 (0.6)	0 (0.0)	
**Rectorrhagia (*N*)**			
No. of episodes per patients (median [IQR])	0 [0,0]	0 [0,0]	0.463
Grade (*N* (%))			
1	11 (7.1)	16 (9.2)	0.6
**Epistaxis (*N*)**			
No. of episodes per patients (median [IQR])	0 [0,0]	0 [0,0]	0.908
Grade (*N* (%))			
0	142 (91.0)	156 (90.2)	
1	13 (8.3)	16 (9.2)	
2	1 (0.6)	1 (0.6)	0.956
**Mucositis (*N*)**			
No. of episodes per patients (median [IQR])	0 [0,2]	0 [0,1]	**0.024**
Grade (*N* (%))			
0	95 (60.9)	124 (71.7)	
1	25 (16.0)	19 (11.0)	0.356
2	26 (16.7)	21 (12.1)	
3	8 (5.1)	7 (4.0)	
4	2 (1.3)	2 (1.2)	
**Neutropenia (*N*)**			
No. of episodes per patients (median [IQR])	0 [0,1]	0 [0,1]	0.691
Grade (*N* (%))			
0	105 (67.3)	115 (66.5)	
1	2 (1.3)	5 (2.9)	0.715
2	22 (14.1)	26 (15.0)	
3	22 (14.1)	19 (11.0)	
4	5 (3.2)	8 (4.6)	
**Fever (*N*)**			
No. of episodes per patients (median [IQR])	0 [0,0]	0 [0,1]	0.647
1	38 (24.4)	49 (28.3)	0.308

*N*, number; *IQR*, interquartile range; %, percentage

In bold: statisticcally significant *p*-values (<0.05)

Moreover, statistically significant differences between males and females were observed in the distribution of the highest toxicity grades of nausea (*p* = 0.024), heartburn (*p* = 0.016), and thrombocytopenia (*p* = 0.034).

In Table 3, the association between sex and presence/absence of toxicity adjusted by age is shown. Females have an increased risk of vomit (OR: 2.057, 95%CI: 1.306–3.238, *p* = 0.002), alopecia (OR: 2.120, 95%CI: 1.053–4.268, *p* = 0.035), heartburn (OR: 1.889, 95%CI: 1.214–2.939, *p* = 0.005), and mucositis (OR: 1.901, 95%CI: 1.241–2.910, *p* = 0.003). Instead, females resulted significantly at lower risk for thrombocytopenia (OR: 0.466, 95%CI: 0.273–0.796, *p* = 0.005).

**Table 3:** Results of logistic regression models on presence/absence of toxicity. Odds ratios are adjusted by age. Reference category is male

Toxicity	OR	95% CI		*p*-value
**Nausea**	1.253	0.943	1.665	0.120
Vomit	2.057	1.306	3.238	**0.002**
Neurotoxicity	0.790	0.559	1.116	0.181
Alopecia	2.120	1.053	4.268	**0.035**
Asthenia	1.147	0.886	1.486	0.297
Diarrhea	0.932	0.701	1.239	0.628
Constipation	1.460	0.860	2.479	0.161
Pain	1.243	0.895	1.726	0.194
Hand-foot syndrome	0.775	0.493	1.218	0.269
Heartburn	1.889	1.214	2.939	**0.005**
Dysgeusia	1.154	0.753	1.769	0.511
Thrombocytopenia	0.466	0.273	0.796	**0.005**
Epistaxis	1.056	0.434	2.566	0.905
Mucositis	1.901	1.241	2.910	**0.003**
Neutropenia	1.029	0.637	1.660	0.908

*OR*, odds ratio; *95% CI*, 95 percent confidence interval

In bold: statisticcally significant *p*-values (<0.05).

### Association between sex and chemotherapy regimen-related toxicities

In Table 4, the association between sex and chemotherapy regimen-related toxicities adjusted by age is reported. Considering topoisomerase inhibitor regimen, females resulted at a significant increase risk of constipation (OR: 1.529, 95%CI: 1.085–2.334), dysgeusia (OR: 1.687, 95%CI: 1.06–2.685), alopecia (OR: 2.243, 95%CI: 1.616–3.112), and heartburn (OR: 3.406, 95%CI: 1.323–8.766). In patients treated with antimetabolite and pyrimidine analogues, females showed an increased risk of vomit (OR: 1.944, 95%CI: 1.207–3.683), constipation (OR: 1.624, 95%CI: 1.281–2.059), alopecia (OR: 2.833, 95%CI: 2.088–3.845), asthenia (OR: 1.24, 95%CI: 1.094–1.406), heartburn (OR: 2.33, 95%CI: 1.401–3.875), and mucositis (OR: 1.891, 95% CI: 1.149–3.114). Among patients treated with DNA binding drugs, females were at higher risk of nausea (OR: 1.466, 95%CI: 1.008–2.131), vomit (OR: 2.422, 95%CI: 1.091–5.375), constipation (OR: 1.814, 95%CI: 1.261–2.609), alopecia (OR: 3.07, 95%CI: 21.706–5.524), asthenia (OR: 1.383, 95%CI: 1.161–1.648), and heartburn (OR: 2.891, 95%CI: 1.577–5.301). Moreover, for both topoisomerase inhibitor and DNA binding groups chemotherapy regimens, females showed a lower risk of thrombocytopenia (OR: 0.002, 95%CI: 0–0.014 and OR: 0.158, 95%CI: 0.031–0.81, respectively). Finally, for growth inhibitor and monoclonal antibody, females showed an increased risk of asthenia (OR: 1.281, 95%CI: 1.066–1.539) and pain (OR: 2.319, 95%CI: 1.282–4.197) events and a lower risk of dysgeusia (OR: 0.703, 95%CI: 0.495–0.999).

**Table 4:** Assessment of sex as a risk factor of toxicity in an analysis stratified by topoisomerase inhibitors, antimetabolite pyrimidine analogues, DNA binding drugs, and growth inhibitor monoclonal antibody chemotherapy regimens

	Topoisomerase inhibitors		Antimetabolite pyrimidine analogues		DNA binding drugs		Growth inhibitor monoclonal antibody	
	OR (95%CI)	*p*	OR (95%CI)	*p*	OR (95%CI)	*p*	OR (95%CI)	*p*
**Nausea**								
Age	0.836 (0.671, 1.041)	0.110	0.861 (0.689, 1.075)	0.187	0.859 (0.685, 1.076)	0.185	0.835 (0.672, 1.039)	0.106
Sex—F:M	1.298 (0.757, 2.225)	0.343	1.27 (0.92, 1.753)	0.146	**1.466 (1.008, 2.131)**	**0.045**	1.016 (0.603, 1.711)	0.952
**Vomit**								
Age	0.703 (0.549, 0.899)	0.116	0.746 (0.5, 1.113)	0.151	0.74 (0.498, 1.1)	0.136	0.734 (0.493, 1.093)	0.128
Sex—F:M	2.181 (0.826, 5.762)	0.123	**1.944 (1.027, 3.683)**	**0.041**	**2.422 (1.091, 5.375)**	**0.030**	1.162 (0.443, 3.046)	0.760
**Constipation**								
Age	**0.760 (0.663, 0.872)**	**< 0.001**	**0.775 (0.675, 0.890)**	**< 0.001**	**0.761 (0.664, 0.872)**	**< 0.001**	**0.759 (0.663, 0.870)**	**< 0.001**
Sex—F:M	**1.592 (1.085, 2.334)**	**0.017**	**1.624 (1.281, 2.059)**	**< 0.001**	**1.814 (1.261, 2.609)**	**0.001**	1.126 (0.769, 1.649)	0.541
**Dysgeusia**								
Age	0.896 (0.792, 1.013)	0.080	0.922 (0.814, 1.044)	0.199	0.931 (0.822, 1.054)	0.258	0.907 (0.802, 1.025)	0.118
Sex—F:M	**1.687 (1.06, 2.685)**	**0.027**	1.15 (0.931, 1.42)	0.20	1.18 (0.907, 1.536)	0.218	**0.703 (0.495, 0.999)**	**0.049**
**Alopecia**								
Age	**1.180 (1.006, 1.382)**	**0.041**	1.145 (0.980, 1.336)	0.087	1.111 (0.952, 1.296)	0.182	**1.168 (1.000, 1.364)**	**0.05**
Sex—F:M	**2.243 (1.616, 3.112)**	**< 0.001**	**2.833 (2.088, 3.845)**	**< 0.001**	**3.07 (1.706, 5.524)**	**< 0.001**	1.066 (0.754, 1.507)	0.716
**Asthenia**								
Age	**1.121 (1.045, 1.203)**	**0.001**	**1.147 (1.069, 1.232)**	**< 0.001**	**1.134 (1.057, 1.216)**	**< 0.001**	**1.107 (1.032, 1.188)**	**0.004**
Sex—F:M	0.843 (0.664, 1.069)	0.159	**1.24 (1.094, 1.406)**	**< 0.001**	**1.383 (1.161, 1.648)**	**< 0.001**	**1.281 (1.066, 1.539)**	**0.008**
**Diarrhea**								
Age	**0.853 (0.775, 0.939)**	**0.001**	**0.866 (0.786, 0.953)**	**0.003**	**0.857 (0.778, 0.943)**	**0.002**	**0.851 (0.773, 0.936)**	**< 0.001**
Sex—F:M	1.191 (0.879, 1.613)	0.259	0.999 (0.841, 1.187)	0.99	0.923 (0.722, 1.181)	0.525	0.797 (0.614, 1.035)	0.089
**Pain**								
Age	1.159 (0.913, 1.473)	0.226	1.173 (0.922, 1.493)	0.194	1.152 (0.908, 1.461)	0.245	1.136 (0.897, 1.437)	0.290
Sex—F:M	1.242 (0.594, 2.597)	0.565	1.406 (0.946, 2.09)	0.09	1.416 (0.772, 2.597)	0.260	**2.319 (1.282, 4.197)**	**0.005**
**Hand-foot syndrome**								
Age	0.822 (0.616, 1.097)	0.184	0.812 (0.612, 1.079)	0.151	0.807 (0.606, 1.073)	0.141	0.838 (0.629, 1.116)	0.226
Sex—F:M	1.289 (0.518, 3.208)	0.585	0.963 (0.575, 1.61)	0.884	0.5 (0.242, 1.031)	0.060	0.676 (0.381, 1.2)	0.181
**Heartburn**								
Age	1.106 (0.794, 1.539)	0.552	1.145 (0.823, 1.591)	0.421	1.121 (0.814, 1.543)	0.484	1.097 (0.794, 1.513)	0.575
Sex—F:M	**3.406 (1.323, 8.766)**	**0.011**	**2.33 (1.401, 3.875)**	**0.001**	**2.891 (1.577, 5.301)**	**< 0.001**	2.043 (0.966, 4.32)	0.062
**Thrombocytopenia**								
Age	**0.645 (0.459, 0.907)**	**0.012**	**0.621 (0.435, 0.887)**	**0.009**	**0.635 (0.443, 0.909)**	**0.013**	**0.638 (0.467, 0.871)**	**0.005**
Sex—F:M	**0.002 (0, 0.014)**	**< 0.001**	0.602 (0.177, 2.049)	0.417	**0.158 (0.031, 0.81)**	**0.027**	0.373 (0.075, 1.841)	0.226
**Epistaxis**								
Age	0.739 (0.404, 1.353)	0.327	0.761 (0.403, 1.437)	0.40	0.730 (0.394, 1.352)	0.317	0.722 (0.389, 1.339)	0.301
Sex—F:M	1.397 (0.329, 5.934)	0.651	1.067 (0.409, 2.783)	0.894	1.052 (0.283, 3.905)	0.940	1.362 (0.454, 4.085)	0.582
**Mucositis**								
Age	0.959 (0.705, 1.304)	0.790	0.980 (0.719, 1.335)	0.896	0.967 (0.710, 1.319)	0.834	0.963 (0.705, 1.315)	0.811
Sex—F:M	1.974 (0.741, 5.26)	0.174	**1.891 (1.149, 3.114)**	**0.012**	1.507 (0.799, 2.843)	0.206	1.659 (0.947, 2.908)	0.077
**Neutropenia**								
Age	0.793 (0.595, 1.056)	0.113	**0.742 (0.556, 0.991)**	**0.043**	0.762 (0.57, 1.019)	0.067	0.803 (0.609, 1.059)	0.120
Sex—F:M	0.735 (0.07, 7.737)	0.798	2.431 (0.465, 12.711)	0.292	2.436 (0.226, 26.219)	0.463	0.298 (0.081, 1.094)	0.068

*OR*, odds ratio; *95% CI*, 95 percent confidence interval

In bold: statisticcally significant *p*-values (<0.05)

### Association between sex and concomitant medication

In the Supplementary Table 2, we described the concomitant medications stratified by sex. Anti-rheumatic agents (1.7% females vs 0 males, *p* = 0.001), hormones, and anti-hormones (2.2% females vs 0.2% males, *p* = 0.001) were more frequently used by females.

## Discussion

Sex differences in CRC prognosis can be explained by pathophysiological differences between males and females and sex specificity of screening tools, which suggests a potential delay in diagnosis for women. Supporting these pieces of evidence, in our analysis, gender-specific differences have been reported.

As observed in our analysis on topoisomerase inhibitor, antimetabolite and pyrimidine analogues, and DNA binding drugs, a different study reported that more women experienced alopecia compared to men when receiving 5-fluorouracil-based treatment [14–21]. Recently, Tejpar and colleagues, evaluating CRC patients undergoing 5-fluorouracyl/leucovorin/irinotecan (FOLFIRI) scheme, observed that baseline neutrophil count, sex, age, poor performance status, and body surface area were associated with an increased incidence of grade III–IV neutropenia; instead, the main predictors for diarrhea were sex and age [22]. A study conducted by Cristina et al., in a population of 2974 CRC patients undergoing FOLFIRI regimen, reported in female population the following toxic events: a higher rates of nausea, as we observed with DNA binding drugs, vomiting, as reported in our patients treated with antimetabolite and pyrimidine analogues and DNA binding drugs regimens, constipation, as observed with topoisomerase inhibitor, antimetabolite and pyrimidine analogues and DNA binding drugs schemes, cramping, stomatitis, cholinergic syndrome, lethargy, alopecia, in line with topoisomerase inhibitor, antimetabolite and pyrimidine analogues and DNA binding drugs in our results, leukopenia, neutropenia, and anemia [23]. All these results were confirmed by the largest study on CRC patients undergoing 5-fluorouracyl single agent (plus folinic acid), with or without oxaliplatin, capecitabine as a single agent or in combination with oxaliplatin and FOLFIRI regimens: female experienced clinically and statistically significant higher toxicity than males, above all severe neutropenia and leukopenia [24]. Evaluating thrombocytopenia, it is already known that women have a larger platelet count than men [25]. A recent Japan study on antibiotic-induced thrombocytopenia reports that there is a higher drug-induced thrombocytopenia in male patients treated with six different drugs, compared with females [26], as we report for topoisomerase inhibitor and DNA binding drugs. In addition, we observed that the female sex is a negative predictor of dysgeusia in those undergoing topoisomerase inhibitor, growth inhibitor, and monoclonal antibody chemotherapy. Female gender is a risk factor for neurosensory disorders in the head and neck, probably due to the sex hormone receptors in the cranial nerve V complex which lead to hypoactivity of the ganglionic inhibitory system [27]. This could be also the explanation for the more frequent pain in female treated with growth inhibitor and monoclonal antibody. In general, there are pieces of evidence that female perceived more pain than males, considering both clinical pain than those reported in animal models [28]. Considering heartburn, while this disease is more common in man [29], we observed a high frequency in a woman undergoing topoisomerase inhibitor, antimetabolite and pyrimidine analogues, and DNA binding drug chemotherapy. Eventually, pieces of evidence in the literature about asthenia and chemotherapy which separately evaluated sexes are lacking; here, we showed a higher percentage of a female with this side effect, during antimetabolite and pyrimidine analogues and DNA binding drugs, growth inhibitor, and monoclonal antibody schemes.

To better tolerate chemotherapy and to reduce the related adverse events, a large use of concomitant drugs is often required. As reported in the literature, sex is one of the several factors able to influence interpatient variability in the drugs dose effect, affecting both pharmacokinetics and pharmacodynamics [5, 30]. In our analysis, anti-rheumatic agents, hormones, and anti-hormones concomitant drugs were more frequently used by a woman (Supplementary table). Coadministered drugs could differently affect chemotherapy response in male and female patients, giving different outcomes and adverse events.

To confirm the reported results, larger prospective studies, incorporating also genetic markers and female hormonal status data, are warranted. Another important limitation of our study was the lack of pharmacokinetic information. Eventually, since we decided to evaluate the single therapeutic agents and not the schemes taken by each patient, the comparison with previous studies was more difficult; a new study protocol, based on a single chemotherapy scheme, is necessary to confirm our results. Individual dose adjustment, based on therapeutic drug monitoring, could lead to significantly improved response rate, survival rate, and toxicities control [31].

## Conclusions

Given the biological and socio-cultural differences between genders, gender-specific analyses should be conducted to provide optimal cancer prevention strategies, to reduce the number of new CRC cases and to better provide treatment both in men and women, a vision that would change the oncology setting of CRC, a stable disease but still orphan of a real tailored approach.

## Supplementary information

Below is the link to the electronic supplementary material.

Supplementary file1 (DOC 138 KB)

Supplementary file2 (DOC 38 KB)
