# Supplementary material for: Colorectal cancer chemotherapy: can sex-specific disparities impact on drug toxicities?
Source: Eur J Clin Pharmacol. 2022 Feb 22;78(6):1029–38. doi: 10.1007/s00228-022-03298-y (PMC9107437; doi:10.1007/s00228-022-03298-y)
Supplement: Supplementary file 1 — Supplementary file1 (DOC 138 KB) [file 228_2022_3298_MOESM1_ESM.doc]

**Table S1. Supplementary table**. Information on treatment schemes.

List of abbreviations: i.a., intravenous administration, o.a., oral administration,  i.a.i.c., intravenous administration by continuous infusion.

| **SCHEME AND DRUGS** | **DOSE** | **U.M.** | **ROUTE OF ADMINISTRATION**  **(interval)** | **DAYS** |
| --- | --- | --- | --- | --- |
| **AFLIBERCEPT+FOLFIRI (one day divided in two)** |  |  |  |  |
| AFLIBERCEPT | 4 mg | mg/kg | i.a. | 1^st^ |
| IRINOTECAN | 180 mg | mg/m^2^ | i.a. | 2^nd^ |
| CALCIUM LEVOFOLINATE | 200 | mg/m^2^ | i.a. | 2^nd^ |
| 5-FLUOROURACIL | 400 | mg/m^2^ | i.a. | 2^nd^ |
| 5-FLUOROURACIL | 2400 | mg/m^2^ | i.a.i.c. (48h) | 2^nd^ |
| **AFLIBERCEPT+FOLFIRI** |  |  |  |  |
| AFLIBERCEPT | 4 mg | mg/kg | i.a. | 1^st^ |
| IRINOTECAN | 180 mg | mg/m^2^ | i.a. | 1^st^ |
| CALCIUM LEVOFOLINATE | 100 | mg/m^2^ | i.a. | 1^st^ |
| 5-FLUOROURACIL | 400 | mg/m^2^ | i.a. | 1^st^ |
| 5-FLUOROURACIL | 1200 | mg/m^2^ | i.a.i.c. (48h) | 1^st^ |
| **AFLIBERCEPT+FOLFIRI (one day)** |  |  |  |  |
| AFLIBERCEPT | 4 mg | mg/kg | i.a. | 1^st^ |
| IRINOTECAN | 180 mg | mg/m^2^ | i.a. | 1^st^ |
| CALCIUM LEVOFOLINATE | 100 | mg/m^2^ | i.a. | 1^st^ |
| 5-FLUOROURACIL | 400 | mg/m^2^ | i.a. | 1^st^ |
| 5-FLUOROURACIL | 2400 | mg/m^2^ | i.a.i.c. (48h) | 1^st^ |
| **BEVACIZUMAB -DE GRAMONT** |  |  |  |  |
| BEVACIZUMAB | 5 mg | mg/kg | i.a. | 1^st^ |
| CALCIUM LEVOFOLINATE | 200 | mg/m^2^ | i.a. | 2^nd^ |
| 5-FLUOROURACIL | 400 | mg/m^2^ | i.a. | 2^nd^ |
| 5-FLUOROURACIL | 2400 | mg/m^2^ | i.a.i.c. (48h) | 2^nd^ |
| **BEVACIZUMAB -DE GRAMONT (two days)** |  |  |  |  |
| BEVACIZUMAB | 5 mg | mg/kg | i.a. | 1^st^ |
| CALCIUM LEVOFOLINATE | 200 | mg/m^2^ | i.a. | 2^nd^, 3^rd^ |
| 5-FLUOROURACIL | 400 | mg/m^2^ | i.a. | 2^nd^, 3^rd^ |
| 5-FLUOROURACIL | 1200 | mg/m^2^ | i.a.i.c. (48h) | 2^nd^ |
| **BEVACIZUMAB (maintenance)** | 7.5 | mg/kg | i.a. | 1^st^ |
| **BEVACIZUMAB +XELODA** |  |  |  |  |
| BEVACIZUMAB | 7.5 mg | mg/kg | i.a. | 1^st^ |
| CAPECITABINE | 35000 |  | o.a. | 1^st^-14^th^ |
| **CARBOPLATIN+ ETOPOSIDE** |  |  |  |  |
| ETOPOSIDE | 1200 | mg/m^2^ | i.a. | 1^st^--3^rd^ |
| CARBOPLATIN | 5 |  | i.a. | 1^st^ |
| **CETUXIMAB MONOCT** |  |  | i.a. |  |
| CETUXIMAB | 400 mg | mg/m^2^ | i.a. | 1^st^ |
| CETUXIMAB | 250 mg | mg/m^2^ | i.a. | 8^th^,15^th^,22^nd^,29^th^,36^th^,43^rd^,50^th^,57^th^,64^th^,71^st^, 78^th^ |
| **CETUXIMAB MONOCT**  **(every 14 days)** | 500 mg | mg/m^2^ | i.a. | 1^st^ , 15^th^, 29^th^, 43^rd^ ,57^th^, 71^st^. |
| **CETUXIMAB +DE GRAMONT** |  |  | i.a. |  |
| CETUXIMAB | 250mg | mg/m^2^ | i.a. | 1^st^-8^th^ |
| CALCIUM LEVOFOLINATE | 200 | mg/m^2^ | i.a. | 2^nd^ |
| 5-FLUOROURACIL | 400 | mg/m^2^ | i.a. | 2^nd^ |
| 5-FLUOROURACIL | 2400 | mg/m^2^ | i.a.i.c. (48h) | 2^nd^ |
| **CETUXIMAB +DE GRAMONT**  **(every 14 days)** |  |  |  |  |
| CETUXIMAB | 500mg | mg/m^2^ | i.a. | 1^st^ |
| CALCIUM LEVOFOLINATE | 200 | mg/m^2^ | i.a. | 2^nd^ |
| 5-FLUOROURACIL | 400 | mg/m^2^ | i.a. | 2^nd^ |
| 5-FLUOROURACIL | 2400 | mg/m^2^ | i.a.i.c. (48h) | 2^nd^ |
| **CETUXIMAB +IRINOTECAN** |  |  |  |  |
| CETUXIMAB | 400mg | mg/m^2^ | i.a. | 1^st^ |
| CETUXIMAB | 250mg | mg/m^2^ | i.a. | 8^th^,15^th^,22^nd^,29^th^,36^th^,43^rd^,50^th^,57^th^,64^th^,71^st^, 78^th^ |
| IRINOTECAN | 180mg | mg/m^2^ | i.a. | 2^nd^ , 16^th^, 30^th^, 44^th^, 58^th^, 72^nd^ |
| **DE GRAMONT** |  |  |  |  |
| CALCIUM LEVOFOLINATE | 100 | mg/m^2^ | i.a. | 1^st^-2^nd^ |
| 5-FLUOROURACIL | 400 | mg/m^2^ | i.a. | 1^st^-2^nd^ |
| 5-FLUOROURACIL | 1200 | mg/m^2^ | i.a.i.c. (48h) | 1^s^ |
| **DE GRAMONT (one day)** |  |  |  |  |
| CALCIUM LEVOFOLINATE | 200 | mg/m^2^ | i.a. | 1^s^ |
| 5-FLUOROURACIL | 400 | mg/m^2^ | i.a. | 1^s^ |
| 5-FLUOROURACIL | 2400 | mg/m^2^ | i.a.i.c. (48h) | 1^s^ |
| **FLO** |  |  |  |  |
| OXALIPLATIN | 85 | mg/m^2^ | i.a. | 1^s^ |
| **FOLFIRI** |  |  |  | 1^s^ |
| IRINOTECAN | 180mg | mg/m^2^ | i.a. | 1^s^ |
| CALCIUM LEVOFOLINATE | 100 | mg/m^2^ | i.a. | 1^st^-2^nd^ |
| 5-FLUOROURACIL | 400 | mg/m^2^ | i.a. | 1^st^-2^nd^ |
| 5-FLUOROURACIL | 1200 | mg/m^2^ | i.a.i.c. (48h) | 1^s^ |
| **FOLFIRI+ BEVACIZUMAB** |  |  |  |  |
| BEVACIZUMAB | 5mg | mg/kg | i.a. | 1^s^ |
| IRINOTECAN | 180mg | mg/m^2^ | i.a. | 1^s^ |
| CALCIUM LEVOFOLINATE | 100 | mg/m^2^ | i.a. | 1^s^ |
| 5-FLUOROURACIL | 400 | mg/m^2^ | i.a. | 1^s^ |
| 5-FLUOROURACIL | 2400 | mg/m^2^ | i.a.i.c. (48h) | 1^s^ |
| **FOLFIRI (one day)** |  |  |  |  |
| IRINOTECAN | 180mg | mg/m^2^ | i.a. | 1^s^ |
| CALCIUM LEVOFOLINATE | 200 | mg/m^2^ | i.a. | 1^s^ |
| 5-FLUOROURACIL | 400 | mg/m^2^ | i.a. | 1^s^ |
| 5-FLUOROURACIL | 2400 | mg/m^2^ | i.a.i.c. (48h) | 1^s^ |
| **FOLFIRI GU+ CETUXIMAB (1 CYCLE)** |  |  |  |  |
| CETUXIMAB | 400 | mg/m^2^ | i.a. | 1^s^ |
| CETUXIMAB | 250 | mg/m^2^ | i.a. | 8^th^ |
| IRINOTECAN | 180mg | mg/m^2^ | i.a. | 1^s^ |
| CALCIUM LEVOFOLINATE | 200 | mg/m^2^ | i.a. | 1^s^ |
| 5-FLUOROURACIL | 400 | mg/m^2^ | i.a. | 1^s^ |
| 5-FLUOROURACIL | 2400 | mg/m^2^ | i.a.i.c. (48h) | 1^s^ |
| **FOLFIRI + CETUXIMAB** **(subsequent cycles)** |  |  |  |  |
| CETUXIMAB | 250 | mg/m^2^ | i.a. | 1^st^-8^th^ |
| IRINOTECAN | 180mg | mg/m^2^ | i.a. | 2^nd^ |
| CALCIUM LEVOFOLINATE | 200 | mg/m^2^ | i.a. | 2^nd^, 3^rd^ |
| 5-FLUOROURACIL | 400 | mg/m^2^ | i.a. | 2^nd^, 3^rd^ |
| 5-FLUOROURACIL | 1200 | mg/m^2^ | i.a.i.c. (48h) | 2^nd^ |
| **FOLFIRI + CETUXIMAB**  **(every 14 days)** |  |  |  |  |
| CETUXIMAB | 500 | mg/m^2^ | i.a. | 1^s^ |
| IRINOTECAN | 180mg | mg/m^2^ | i.a. | 2^nd^ |
| CALCIUM LEVOFOLINATE | 200 | mg/m^2^ | i.a. | 2^nd^ |
| 5-FLUOROURACIL | 400 | mg/m^2^ | i.a. | 2^nd^ |
| 5-FLUOROURACIL | 2400 | mg/m^2^ | i.a.i.c. (48h) | 2^nd^ |
| **FOLFOX 4** |  |  |  |  |
| OXALIPLATIN | 85 | mg/m^2^ | i.a. | 1^s^ |
| CALCIUM LEVOFOLINATE | 200 | mg/m^2^ | i.a. | 2^nd^ |
| 5-FLUOROURACIL | 400 | mg/m^2^ | i.a. | 1^st^-2^nd^ |
| 5-FLUOROURACIL | 2400 | mg/m^2^ | i.a.i.c. (48h) | 2^nd^ |
| **FOLFOX 6** |  |  |  |  |
| OXALIPLATIN | 100 | mg/m^2^ | i.a. | 1^s^ |
| CALCIUM LEVOFOLINATE | 200 | mg/m^2^ | i.a. | 1^s^ |
| 5-FLUOROURACIL | 400 | mg/m^2^ | i.a. | 1^s^ |
| 5-FLUOROURACIL | 2400 | mg/m^2^ | i.a.i.c. (22h) | 1^s^ |
| **FOLFOX 6+BEVACIZUMAB** |  |  |  |  |
| BEVACIZUMAB | 5 | mg/kg | i.a. | 1^s^ |
| OXALIPLATIN | 100 | mg/m^2^ | i.a. | 2^nd^ |
| CALCIUM LEVOFOLINATE | 200 | mg/m^2^ | i.a. | 2^nd^ |
| 5-FLUOROURACIL | 400 | mg/m^2^ | i.a. | 2^nd^ |
| 5-FLUOROURACIL | 2400 | mg/m^2^ | i.a.i.c. (48h) | 2^nd^ |
| **FOLFOX 6+CETUXIMAB (1 CYCLE)** |  |  |  |  |
| CETUXIMAB | 500 | mg/kg | i.a. | 1^s^ |
| OXALIPLATIN | 100 | mg/m^2^ | i.a. | 2^nd^ |
| CALCIUM LEVOFOLINATE | 200 | mg/m^2^ | i.a. | 2^nd^ |
| 5-FLUOROURACIL | 400 | mg/m^2^ | i.a. | 2^nd^, 3^rd^ |
| 5-FLUOROURACIL | 2400 | mg/m^2^ | i.a.i.c. (48h) | 2^nd^ |
| **FOLFOX 6+CETUXIMAB (subsequent cycles)** |  |  |  |  |
| CETUXIMAB | 250 | mg/kg | i.a. | 1^st^-8^th^ |
| OXALIPLATIN | 85 | mg/m^2^ | i.a. | 2^nd^ |
| CALCIUM LEVOFOLINATE | 200 | mg/m^2^ | i.a. | 3 |
| 5-FLUOROURACIL | 400 | mg/m^2^ | i.a. | 2^nd^, 3^rd^ |
| 5-FLUOROURACIL | 1200 | mg/m^2^ | i.a.i.c. (48h) | 2^nd^ |
| **FOLFOXIRI** |  |  |  |  |
| IRINOTECAN | 130 | mg/kg | i.a. | 1^s^ |
| OXALIPLATIN | 85 | mg/m^2^ | i.a. | 1^s^ |
| CALCIUM LEVOFOLINATE | 200 | mg/m^2^ | i.a. | 1^s^ |
| 5-FLUOROURACIL | 1200 | mg/m^2^ | i.a.i.c. (48h) | 2^nd^ |
| **FOLFOXIRI+ BEVACIZUMAB** |  |  |  |  |
| **BEVACIZUMAB** | 5 | mg/kg | i.a. | 1^s^ |
| IRINOTECAN | 165 | mg/m^2^ | i.a. | 2^nd^ |
| OXALIPLATINO | 85 | mg/m^2^ | i.a. | 2^nd^ |
| CALCIO LEVOFOLINATO | 200 | mg/m^2^ | i.a. | 2^nd^ |
| 5-FLUOROURACILE | 3200 | mg/m^2^ | i.a.i.c. (48h) | 2^nd^ |
| **IRINOTECAN Q15** |  |  |  |  |
| IRINOTECAN | 180 | mg/m^2^ | i.a. | 1^st^-8^th^-15^th^ |
| **OXAPETRELLI** |  |  |  |  |
| OXALIPLATINO | 50 | mg/m^2^ | i.a. | 1^st^-8^th^-15^th^ |
| 5-FLUOROURACILE | 500 | mg/m^2^ | i.a. | 1^st^-8^th^-15^th^ |
| **PACLITAXEL weekly** |  |  |  |  |
| PACLITAXEL | 80 | mg/m^2^ | i.a. | 1^st^-8^th^-15^th^ |
| **PANITUMUMAB** |  |  |  |  |
| PANITUMUMAB | 6 | mg/kg | i.a. | 1^s^ |
| **PANITUMUMAB+ FOLFOX 4** |  |  |  |  |
| PANITUMUMAB | 6 | mg/kg | i.a. | 1^s^ |
| OXALIPLATINO | 85 | mg/m^2^ |  | 2^nd^ |
| **PANITUMUMAB+ FOLFOX 6** |  |  |  |  |
| PANITUMUMAB | 6 | mg/kg | i.a. | 1^s^ |
| OXALIPLATINO | 100 | mg/m^2^ | i.a. | 2^nd^ |
| CALCIO LEVOFOLINATO | 200 | mg/m^2^ | i.a. | 2^nd^ |
| 5-FLUOROURACILE | 400 | mg/m^2^ | i.a. | 2^nd^ |
| 5-FLUOROURACILE | 2400 | mg/m^2^ | i.a.i.c. (48h) | 2^nd^ |
| **PETRELLI** |  |  |  |  |
| 5-FLUOROURACILE | 500 | mg/m^2^ | i.a. | 1^st^-8^th^-15^th^ |
| **RALTITREXED** |  |  |  |  |
| RALTITREXED | 3 | mg/m^2^ | i.a. | 1^s^ |
| **TOMIRI** |  |  |  |  |
| RALTITREXED | 3 | mg/m^2^ | i.a. | 1^s^ |
| IRINOTECAN | 240 | mg/m^2^ | i.a. | 1^s^ |
| **TOMOX** |  |  |  |  |
| RALTITREXED | 3 | mg/m^2^ | i.a. | 1^s^ |
| OXALIPLATINO | 130 | mg/m^2^ | i.a. | 1^s^ |
| **XELIRI** |  |  |  |  |
| IRINOTECAN | 200 | mg/m^2^ | i.a. | 1^s^ |
| CAPECITABINA | 28000 |  | o.a. | 1^st^-14^th^ |
| **XELIRI+ BEVACIZUMAB** |  |  |  |  |
| BEVACIZUMAB | 7.5 | mg/kg | i.a. | 1^s^ |
| IRINOTECAN | 200 | mg/m^2^ | i.a. | 2^nd^ |
| CAPECITABINE | 28000 |  | o.a. | 1^st^-14^th^ |
| **XELODA** |  |  |  |  |
| CAPECITABINE | 28000 |  | o.a. | 1^st^-14^th^ |
| **XELOX+ BEVACIZUMAB** |  |  |  |  |
| BEVACIZUMAB | 7,5 | mg/kg | i.a. | 1^s^ |
| OXALIPLATIN | 130 | mg/m^2^ | i.a. | 2^nd^ |
| CAPECITABINE | 28000 |  | o.a. | 1^st^-14^th^ |
| **XELOX+ CETUXIMAB (1 CYCLE)** |  |  |  |  |
| CETUXIMAB | 400 | mg/m^2^ | i.a. | 1^s^ |
| CETUXIMAB | 250 | mg/m^2^ | i.a. | 8-15g |
| OXALIPLATIN | 130 | mg/m^2^ | i.a. | 2^nd^ |
| CAPECITABINE | 28000 |  | o.a. | 1^st^-14^th^ |
| **XELOX+ CETUXIMAB weekly** |  |  |  |  |
| CETUXIMAB | 250 | mg/m^2^ |  | 1^st^-8^th^-15^th^ |
| OXALIPLATIN | 130 | mg/m^2^ |  | 2^nd^ |
| CAPECITABINE | 28000 |  | o.a. | 1^st^-14^th^ |
| **XELOX** |  |  |  |  |
| OXALIPLATIN | 50 | mg/m^2^ | i.a. | 1^s^ |
| CAPECITABINE | 23100 |  | o.a. | 1^st^-14^th^ |
