# Supplementary material for: Colorectal cancer chemotherapy: can sex-specific disparities impact on drug toxicities?
Source: Eur J Clin Pharmacol. 2022 Feb 22;78(6):1029–38. doi: 10.1007/s00228-022-03298-y (PMC9107437; doi:10.1007/s00228-022-03298-y)
Supplement: Supplementary file 2 — Supplementary file2 (DOC 38 KB) [file 228_2022_3298_MOESM2_ESM.doc]

**Table S2. Supplementary table**. Description of concomitant medication.

List of abbreviations: N, number; %, percentage.

| **Concomitant medication** | **F**  **N (%)** | **M**  **N (%)** | | ***p-value*** |
| --- | --- | --- | --- | --- |
|  |  |  |  | |
| Diuretics | 31 (4.9) | 48 (6.0) | 0.447 | |
| Beta blockers | 27 (4.3) | 53 (6.6) | 0.074 | |
| Ivabradine and ranolazine | 6 (1.0) | 4 (0.5) | 0.482 | |
| Antiarrhythmics | 0 (0.0) | 2 (0.2) | 0.588 | |
| Antiplatelets, anticoagulants and fibrinolytics | 40 (6.3) | 70 (8.7) | 0.114 | |
| Opioid analgesics | 90 (14.3) | 120 (14.9) | 0.774 | |
| NSAID (nonsteroidal anti-inflammatory drug) | 85 (13.5) | 113 (14.1) | 0.802 | |
| Drugs acting on the central nervous system | 47 (7.4) | 62 (7.7) | 0.926 | |
| Lipid-lowering agents | 11 (1.7) | 16 (2.0) | 0.882 | |
| Antihyperglycaemics | 13 (2.1) | 27 (3.4) | 0.185 | |
| Drug treatment for prostatic hyperplasia | 0 (0.0) | 12 (1.5) | 0.005 | |
| Corticosteroids | 72 (11.4) | 83 (10.3) | 0.572 | |
| Iron and vitamins supplements | 86 (13.6) | 107 (13.3) | 0.929 | |
| Antiemetics | 97 (15.4) | 95 (11.8) | 0.061 | |
| Laxatives | 94 (14.9) | 113 (14.1) | 0.715 | |
| Eyedrops for glaucoma | 3 (0.5) | 3 (0.4) | 1.000 | |
| Muscle relaxers | 1 (0.2) | 0 (0.0) | 0.904 | |
| Bronchodilators and mucolytics | 7 (1.1) | 7 (0.9) | 0.854 | |
| Vasoprotective agents | 0 (0.0) | 6 (0.7) | 0.078 | |
| Vasoconstriction agents | 0 (0.0) | 1 (0.1) | 1.000 | |
| Anticholinergics | 6 (1.0) | 5 (0.6) | 0.688 | |
| Immunosuppressive drugs | 0 (0.0) | 1 (0.1) | 1.000 | |
| Cytokine | 20 (3.2) | 13 (1.6) | 0.077 | |
| Bisphosphonates | 3 (0.5) | 0 (0.0) | 0.170 | |
| Antigout | 3 (0.5) | 7 (0.9) | 0.565 | |
| Antihistamines | 30 (4.8) | 49 (6.1) | 0.320 | |
| Bile acid sequestrants | 2 (0.3) | 1 (0.1) | 0.834 | |
| Anti-rheumatic agents | 11 (1.7) | 0 (0.0) | **0.001** | |
| Hormones and anti-hormones | 14 (2.2) | 2 (0.2) | **0.001** | |
| Proton-pump inhibitors and gastro-protective agents | 126 (20.0) | 135 (16.8) | 0.142 | |
| Moisturizing, healing, antibiotic, anesthetic creams | 63 (10.0) | 84 (10.5) | 0.835 | |
| Antimicrobial agents | 82 (13.0) | 129 (16.1) | 0.120 | |
| Antifungals | 48 (7.6) | 57 (7.1) | 0.791 | |
| Cytostatic agents | 9 (1.4) | 6 (0.7) | 0.321 | |
| Disinfettanti = 1 (%) | 1 (0.2) | 0 (0.0) | 0.904 | |
| Disinfectant agents | 10 (1.6) | 8 (1.0) | 0.450 | |
| Antivirals | 0 (0.0) | 2 (0.2) | 0.588 | |
| Parenteral nutrition | 9 (1.4) | 6 (0.7) | 0.321 | |
| Anti-cystitis treatments | 0 (0.0) | 1 (0.1) | 1.000 | |
| Manittol | 631 (100.0) | 803 (100.0) | NA | |
| Dihydropyridine derivatives, alpha1-blockers and ace-inhibitors | 67 (10.6) | 98 (12.2) | 0.395 | |
